# Occupational stress in primary care workers during the COVID-19 pandemic: mixed methods study

**DOI:** 10.1590/1518-8345.6797.4041

**Published:** 2023-11-03

**Authors:** Marcilene Marques de Freitas Tamborini, Alexa Pupiara Flores Coelho Centenaro, Eliane Nogueira de Souza Souto, Alana Thais Gisch Andres, Eniva Miladi Fernandes Stumm, Christiane de Fátima Colet

**Affiliations:** 1 Universidade Regional do Noroeste do Estado do Rio Grande do Sul, Núcleo de Ciências da Saúde, Ijuí, RS, Brasil.; 2 Universidade Federal de Santa Maria, Departamento de Enfermagem, Palmeira das Missões, RS, Brasil.; 3 Universidade Brasil, Núcleo de Ciências da Saúde, Fernandópolis, SP, Brasil.

**Keywords:** Health Personnel, Occupational Stress, Primary Health Care, COVID-19, Occupational Health, Mental Health, Personal de Salud, Estrés Laboral, Atención Primaria de Salud, COVID-19, Salud Laboral, Salud Mental, Profissional de Saúde, Estresse Ocupacional, Atenção Primária à Saúde, COVID-19, Saúde do Trabalhador, Saúde Mental

## Abstract

**Objective:**

to analyze the risk of exposure to occupational stress among primary healthcare professionals during the COVID-19 pandemic and their perception regarding their experience.

**Method:**

mixed-methods sequential explanatory study with 50 primary care professionals. Sociodemographic, clinical, and labor questionnaires, Job Stress Scale, and semi-structured interviews were used. Quantitative data were submitted to descriptive and analytical statistical analysis; qualitative data were submitted to Thematic Content Analysis.

**Results:**

66% of professionals were exposed to occupational stress. Doctors were associated with highly demanding work (p<0.001); nurses, nursing technicians, and dental professionals with active work (p<0.001); and dentists with lower psychological demand (p<0.001). Professionals with more than sixteen years of experience had better conditions to deal with stressful factors, compared to those with less than five years (p<0.03). Data integration showed implications of the pandemic in life, work, and interfaces with psychological symptoms.

**Conclusion:**

professionals worked under high psychological demands and a high risk of exposure to stress during the COVID-19 pandemic. Self-control and high social support may contribute to reducing these risks, as well as professional training and experience.

## Abstract (pt)

**Objetivo:**

analisar o risco de exposição ao estresse ocupacional em profissionais de saúde da Atenção Primária à Saúde durante a pandemia da COVID-19 e sua percepção sobre essa vivência.

**Método:**

estudo de métodos mistos do tipo explanatório sequencial, com 50 profissionais da atenção primária. Foram utilizados questionários sociodemográfico, clínico e laboral, *Job Stress Scale* e entrevista semiestruturada. Os dados quantitativos foram submetidos à análise estatística descritiva e analítica; os qualitativos, à Análise Temática de Conteúdo.

**Resultados:**

66% dos profissionais apresentaram exposição ao estresse ocupacional. A profissão médica associou-se ao trabalho de alta exigência (p<0,001); enfermeiros, técnicos em Enfermagem, profissionais da Odontologia, ao trabalho ativo (p<0,001); dentistas, a menor demanda psicológica (p<0,001). Profissionais com mais de dezesseis anos de formados apresentaram melhores condições para lidar com fatores estressantes, comparados aos com menos de cinco anos (p<0,03). A integração dos dados evidenciou implicações da pandemia na vida, no trabalho e interfaces com os sintomas psicológicos.

**Conclusão:**

os profissionais trabalharam sob altas demandas psicológicas e elevado risco de exposição ao estresse durante a pandemia pela COVID-19. Autocontrole e elevado apoio social podem contribuir para redução desses riscos, assim como tempo de formação e experiência profissional.

## Abstract (es)

**Objetivo:**

analizar el riesgo de exposición al estrés laboral de los profesionales de la Atención Primaria de la Salud durante la pandemia de COVID-19 y la percepción que tienen sobre esa experiencia.

**Método:**

estudio de métodos mixtos del tipo explicativo secuencial, con 50 profesionales de la atención primaria. Se utilizaron cuestionarios sociodemográficos, clínicos y laborales, *Job Stress Scale* y entrevista semiestructurada. Los datos cuantitativos fueron sometidos a análisis estadístico descriptivo y analítico; los cualitativos, a Análisis de Contenido Temático.

**Resultados:**

el 66% de los profesionales estuvieron expuestos a estrés laboral. La profesión médica se asoció al trabajo de alta exigencia (p<0,001); los enfermeros, técnicos en enfermería, profesionales de odontología, al trabajo activo (p<0,001); los odontólogos, a menor exigencia psicológica (p<0,001). Los profesionales con más de dieciséis años de graduados presentaron mejores condiciones para lidiar con los estresores que aquellos con menos de cinco años (p<0,03). La integración de datos demostró que la pandemia repercutió en la vida, el trabajo e interfaces con los síntomas psicológicos.

**Conclusión:**

los profesionales trabajaron bajo altas exigencias psicológicas y alto riesgo de exposición al estrés durante la pandemia de COVID-19. El autocontrol y un alto apoyo social pueden contribuir a reducir estos riesgos, así como el tiempo de formación y la experiencia profesional.

**Highlights**

**(1)** Primary Health Care professionals work under high psychological demand.

**(2)** They are exposed to a high risk of occupational stress and psychological symptoms.

**(3)** Social support exerts great influence to minimize risks to mental health.

**(4)** The work in the pandemic contributed to increase psychological symptoms in the worker.

**(5)** Professional experience and length of service minimized risk factors in the pandemic.

## Introduction

The coronavirus disease 2019 (COVID-19) pandemic, among healthcare workers has affected those who contracted the disease as well as those who remained physically healthy ^(^ 1 ^)^. The impacts of the pandemic have gone beyond physical illness, causing psychosocial stress, with an effect on quality of life ^(^ 2 ^)^. 

The pandemic scenario contributed to the increase of the already existing deficiencies in the workflow of health units and exposed health professionals to new stressors ^(^ 3 ^)^, making them one of the most susceptible groups to psychological distress, when compared to the general population ^(^ 4 ^-^ 5 ^)^. This panorama suggests the need for greater care for their mental health. 

Some national and international studies have reported psychological symptoms in health professionals during the COVID-19 pandemic. Scientific evidence has shown high rates of psychological distress in the work environment during the pandemic, triggering symptoms of anxiety, depression, and stress ^(^ 6 ^-^ 8 ^)^. 

Work-related stress has increased among health professionals, especially in times of crisis, such as pandemics ^(^ 2 ^-^ 3 ^)^. Although studies have investigated the psychological impacts on health workers during these times, most research has focused on hospital environments ^(^ 7 ^,^ 9 ^-^ 10 ^)^, and few national and international studies focus on Primary Health Care (PHC). Given that the presence of stress associated with working conditions has affected these professionals’ mental health, in addition to being a facilitating agent for other diseases. It is necessary to better understand these scenarios, seeking to identify risk factors in order to prevent the occurrence of stress and reduce the occurrence of health problems among these professionals. This highlights this study’s innovative potential and the relevance of seeking a greater understanding of the work and health context of professionals involved in PHC care in the pandemic scenario. 

In view of these considerations, this study aimed to analyze the risk of exposure to occupational stress in PHC health professionals during the COVID-19 pandemic and their perception regarding this experience.

## Method

### Study type

This is a mixed-methods study, which comprises the integration of quantitative and qualitative sources of evidence to produce in-depth knowledge about a complex research problem. A sequential explanatory design was employed, in which a quantitative stage is carried out, followed by a subsequent qualitative stage. This design allows qualitative evidence to be used to deepen or explain quantitative findings ^(^ 11 ^)^. In this study, greater weight was given to the quantitative analysis (a correlational cross-sectional study) and lesser weight to the qualitative analysis (a descriptive qualitative study), thus being represented by the form QUAN ➝ Qual. 

### Site

It was developed in the PHCU of a municipality in southern Brazil, which has four Primary Health Care Units (PHCU) and 18 Family Health Strategies (FHS); all participated in the study.

### Time period

Quantitative data collection took place between June and September 2021, and qualitative data between January and February 2022.

### Population and selection criteria

The study was conducted with a population of 162 PHC health professionals, including doctors, nurses, nursing technicians, nutritionists, and dental professionals. Due to the fact that it was a viable population for the study design, it was decided to work with the entire eligible population, using the response rate as a sampling parameter.

The eligibility criteria were met by health professionals: doctors, nutritionists, and nursing and dentistry professionals, who provided care at these units during the pandemic. Those who were on vacation or on leave during data collection were excluded.

Therefore, when applying the eligibility criteria, five professionals who were on vacation and 18 who were on leave during the data collection periods were excluded. In addition, 32 professionals refused (alleging other activities and lack of time) and 57 professionals did not respond to the four attempts to contact them in person and online. Thus, 50 participated, i.e., 32.4% of the total proposed universe.

### First phase: instruments and quantitative data collection

In this study, greater value was given to the quantitative stage, which was developed in the first instance. Data were collected through a self-applicable online instrument built in Google Forms containing: sociodemographic, work, and clinical questionnaires; questions related to COVID-19; and a work stress scale.

The questionnaire included socio-occupational and clinical data prepared by the researchers with the following variables: gender, age, marital status, children, education, postgraduate studies, position, unit of assignment, workload, working hours, training time, physical activity, leisure, health problems, medication use and absence from work. It also contained questions regarding COVID-19 related to contingency measures, contamination, and medication use.

The Job Stress Scale (JSS) was used in a version translated and adapted for the Portuguese language ^(^ 12 ^)^. The Demand-Control Model (DCM) was used to assess exposure to occupational stress, based on the JSS, which evaluates psychosocial factors and risk of stress in work activities. The scale consists of a self-applicable questionnaire, containing 17 questions, divided into three dimensions: psychological demand (five questions), control (six questions), and social support (six questions) ^(^ 12 ^)^. 

Items are measured by scores on a four-point Likert scale, ranging from often to never/almost never or from strongly agree to strongly disagree. For the demand and control questions, the score ranges from 1 (never or almost never) to 4 (often); for the social support questions, the score ranges from 4 (strongly agree) to 1 (strongly disagree). For questions 4 (“Do you have enough time to accomplish all the tasks in your job?”) and 9 (“In your job, do you have to repeat the same task many times?”) the analyses were reversed. In each dimension of the scale, the higher the score, the greater the perceived demand, control, or social support.

For the dichotomy, in the JSS bivariate statistical analysis, the cut-off point is the total score median of each dimension. The “psychological demand” domain score, which ranges from five to 20 points, was dichotomized into low demand (five to 14 points) and high demand (15 to 20 points). The “control over work” dimension score, ranges from six to 24 points and was dichotomized by the median into low control (nine to 17 points) and self-control (18 to 24 points). The score of the “social support” domain ranges from six to 24 points and was dichotomized into low social support (six to 10 points) and high social support (11 to 24 points) ^(^ 12 ^)^. Finally, the concept of the DCM quadrants was stratified into low demand (self-control and low demand), passive work (low control and low demand), active work (self-control and high demand), and high demand (low control and high demand). The “social support” dimension acts as a stress mediator in the work environment ^(^ 12 ^)^. 

The instrument was sent to the study population through institutional e-mails and messaging applications. In the second phase, a face-to-face/in-person data collection was conducted, seeking to access the professionals who had not responded online. The questionnaires were delivered to the participants in the units, after the presentation and explanation of the study objectives.

### Second phase: instrument and qualitative data collection

In this study, less weight was given to the qualitative stage, which was developed at a later stage. This stage was planned following an initial analysis of the quantitative data. Therefore, the objective was to generate subjective data that would contribute to elucidating the relationship between stress and health work during the COVID-19 pandemic. To this end, individual semi-structured interviews were conducted with a sample of 14 professionals (doctors, nutritionists, nurses, nursing technicians, and dentists). The professionals were selected through a simple random draw with those who participated in the quantitative stage. Thus, the qualitative stage was characterized as an unfolding of the quantitative stage.

The number of participants was established using the criterion of theoretical saturation of the data ^(^ 13 ^)^. Thus, the collection was interrupted with the achievement of 14 interviews, as sufficient results were considered obtained for this stage. 

To conduct the interviews, a script prepared by the researchers was used, addressing the topics: stressful factors at work during the pandemic; feelings experienced; support received; difficulties, and challenges faced. This script was planned considering the need to obtain qualitative findings that would contribute to explaining/deepening the quantitative findings. Therefore, investigating these elements was important in order to build a qualitative database whose content would enable inferences to be drawn which could shed light on the quantitative findings.

The interviews were conducted online by the lead researcher, previously trained, for an average of 35 minutes. The statements were transcribed by a team of transcribers from the research group of the Federal University of Santa Maria, previously trained for this task. After the transcriptions, checking and double-checking were performed to check for possible errors and strengthen data reliability. The full interview transcriptions comprised the qualitative corpus for analysis.

### Data processing and analysis

Quantitative data were entered into the Microsoft Excel ^®^ program by two independent typists. After checking for possible errors and/or inconsistencies, the data were transferred to the Statistical Package for the Social Sciences (SPSS) software, version 22.0, and analyzed with descriptive and inferential statistics. Descriptive statistics were used to characterize the participants’ sociodemographic, work, and clinical variables. Quantitative variables were described by measures of central tendency and dispersion. The chi-square test and Fisher’s exact test were used for the association and/or correlation between variables, with p-values < 0.05 considered significant. 

The qualitative data followed the Thematic Content Analysis through three stages: pre-analysis, material exploration, and data treatment/interpretation. The pre-analysis corresponds to the researcher’s first interactions with the empirical material ^(^ 14 ^)^. A floating reading was performed in order to better understand and appropriate the content. After reading and re-reading the material, it was refined and organized, highlighting the statements that contributed to the understanding of the object of study. 

Material exploration consists of a long phase, through which the emerging themes are identified, decomposed, and coded into Registration Units (URs) ^(^ 14 ^)^. Five URs were extracted and coded (anxiety, stress, insomnia, fear/insecurity, and vulnerability). The URs were organized by semantic affinity into two analytical axes: Demand, control, and social support of PHC professionals in the COVID-19 pandemic and Self-perceived stress by PHC professionals during the COVID-19 pandemic: interfaces with demand, control, and social support. 

Finally, in data processing and interpretation, with significant and faithful results, the researcher can make inferences and advance interpretations according to the study objective ^(^ 14 ^)^. This step was conducted in parallel with the data mixing process. That is, the interpretation of qualitative findings occurred from the interface with quantitative ones, seeking similarities, complementarity, or divergences. 

### Data integration

The integration between quantitative and qualitative findings occurred according to the specific framework of mixed methodology ^(^ 11 ^-^ 15 ^)^. In accordance with the sequential explanatory design, an explanatory integration was performed, that is, the qualitative findings were used to enlighten the quantitative ones, in an explanatory sequential design ^(^ 15 ^)^. To this end, inferences and narratives were sought in the statements that helped to contextualize the experience of stress, enlightening the experiences related to it and the possible interfaces with the pandemic scenario (central context of this study). 

In order to make this integration feasible, the tools called joint-displays, which are visual representations illustrating the QUAN➝Qual integration, and meta-inferences, which visually represent the integration in mixed-method designs ^(^ 15 ^)^, were employed. For this purpose, the software CmapTools version 6.04 was used, a tool that enables the building of mind and concept maps. 

In the presentation of the results, there are excerpts from the participants’ testimonies. The interviewees are identified by the letter P (“professional”) followed by a random cardinal number.

### Ethical aspects

The project was approved by the National Research Ethics Committee, with protocol number 30792920.5.1001.5350.

## Results

Among the 50 participants, 88% were female; 42.0% were nurses and 26.0% were nursing technicians. Doctors, nutritionists, and dental professionals had a lower percentage. Most were married (60.0%), with children (74.0%), and aged between 41 and 50 years (38.0%).

Regarding labor characteristics, 54.0% worked eight hours a day and 50.0% worked 40 hours a week. Also, 84.0% reported having a single employment contract; 48.0% reported having more than 16 years of experience, as shown in Table 1. 

**Table 1 -:** Sociodemographic and work characterization of health professionals in primary care during the COVID-19 pandemic (*n=50). Ijuí, RS, Brazil, 2021

Variables		n*	% ^†^
Sex	Female	44	88.0
	Male	6	12.0
Age group	18 to 30 years	10	20.0
	31 to 40 years	16	32.0
	Over 41 years old	24	48.0
Marital status	Married/stable union	40	80.0
	Single/separated	10	20.0
Has children	Yes	37	74.0
	No	13	26.0
Daily working hours	8 hours	27	54.0
	12 hours	22	44.0
	Other	1	2.0
Weekly working hours	36 hours	3	6.0
	40 hours	25	50.0
	44 hours	2	4.0
	Other	20	40.0
Has another employment contract	No	42	84.0
	Yes	8	16.0
Length of education	Less than 1 year to 10 years	14	28.0
	11 to 15 years	12	24.0
	More than 16 years	24	48.0
Postgraduate degree	Yes	24	48.0
	No	26	52.0
Total		50	100

*n = Sample; ^†^Percentage

In the qualitative stage, among the 14 interviewees, there was a predominance of women (n=12, 85.7%), nurses (n=8, 57.1%), and an average age between 31-40 years (50.0%).

**Figure 1 -:**
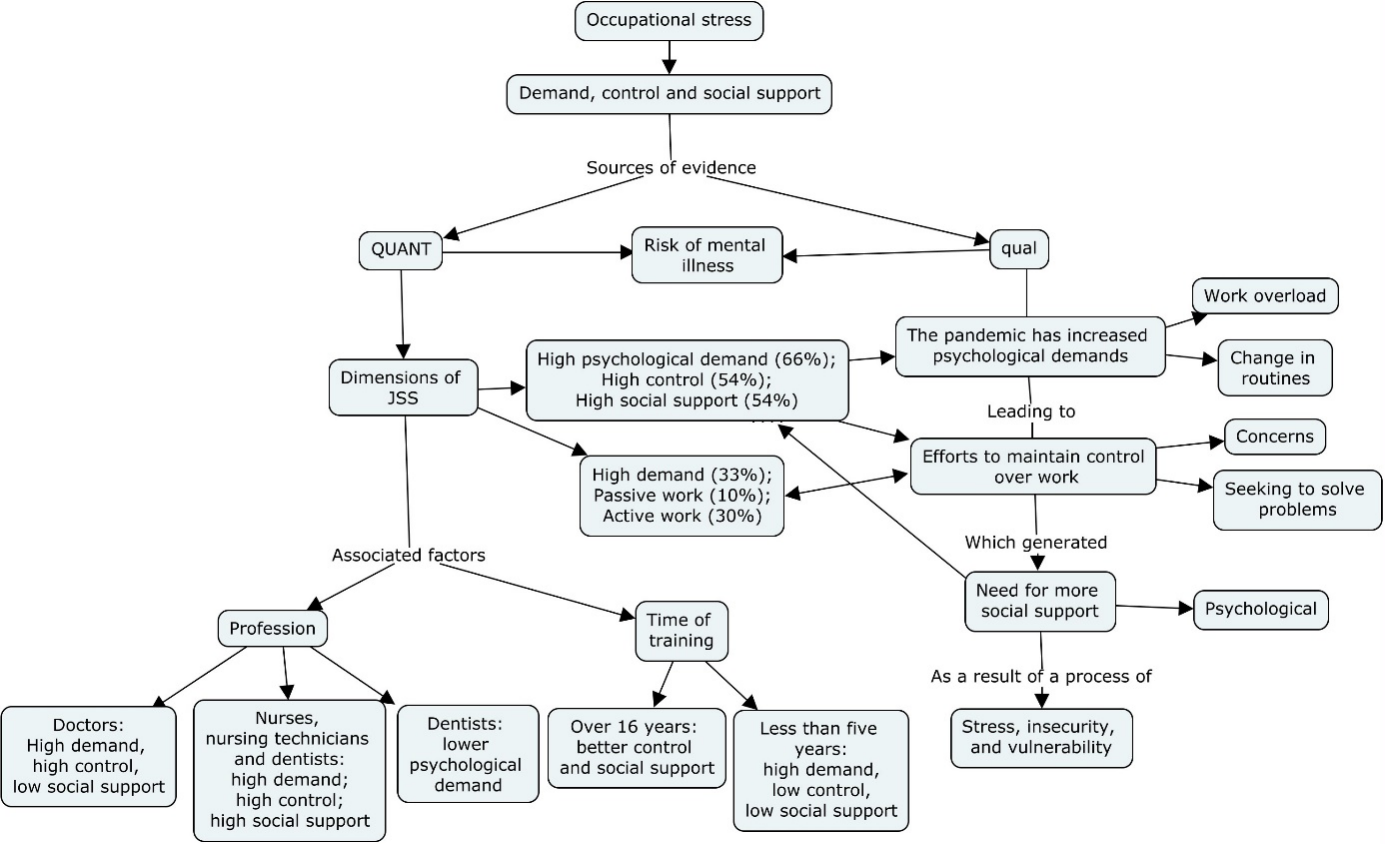
Joint-display illustrating the QUAN➝Qual integration and meta-inference of findings related to occupational stress (demand, control, and social support) of PHC professionals during the COVID-19 pandemic. Ijuí, RS, Brazil, 2021

Source: Designed by the authors using CmapTools 6.04 software

Figure 1 illustrates the representative joint-display of the QUAN➝Qual data integration. Next, both axes of analysis that discuss the findings of the study will be presented. The first was called: Demand, control, and social support of PHC professionals during the COVID-19 pandemic; and the second: Self-perceived stress by PHC professionals during the COVID-19 pandemic: interfaces with demand, control, and social support. 

### Demand, control, and social support of PHC professionals during the COVID-19 pandemic

Sociodemographic and occupational characteristics (independent variables) were associated with the dimensions of JSS (dependent variables). The medical profession was associated with high demand, lower control, and low social support (p<0.001); nurses, nursing technicians and dental professionals with high demand, high control, and high social support (p<0.001); dentists were associated with lower psychological demand (p<0.001). Professionals with more than 16 years of experience showed better scores in the control and social support dimensions, while professionals with less than five years of experience showed high demand, low control, and low social support (p<0.03), as detailed in Table 2

**Table 2 -:** Labor characteristics of health professionals working in primary care during the COVID-19 pandemic according to the dimensions of the Job Stress Scale (*n=50). Ijuí, RS, Brazil, 2021

Variables		Demand				Control		Social support	
		n*	% ^†^	Low	High	Low	High	Low	High
Occupation	Nurse	21	42.0	10(47.6)	11(52.4)	10(47.6)	11(52.4)	7(33.3)	14(66.7)
	Nursing Technician	13	26.0	3 (23.1)	10(76.9)	4(30.8)	9(69.2)	5(38.5)	8(61.5)
	Doctor	11	22.0	3 (23.7)	8(72.7)	7(63.6)	4(36.4)	10(90.9)	1(9.1)
	Others ^§^	5	10.0	1(10.0)	4(40.0)	2(20.0)	3(30.0)	1(50.0)	4(40.0)
				p<0.001 ^‡^		p<0.001 ^‡^		p<0.001 ^‡^	
Time since graduation	Less than 1 year	14	28.0	4(28.5)	10(71.4)	9 (64.2)	5(35.7)	9(64.2)	5(35.7)
	11 to 15 years	12	24.0	4(33.3)	8(66.7)	8(66.7)	4(33.3)	7(58.3)	5(41.7)
	More than 16 years	24	48.0	9(37.5)	15(62.5)	6(25.0)	18(75.0)	7(29.2)	17 (70.8)
				p<0.93 ^‡^		p<0.03 ^‡^		p<0.07 ^‡^	
	Total	50	100	17(34.0)	33(66.0)	23(46.0)	27(54.0)	23(46.0)	27(54.0)

*n = Sample; ^†^Percentage; ^‡^Chi-square test, significant for p<0.05; ^§^Others = Nutritionist, dentist, and dental assistant categories had an n value between 2 and 1 and were grouped in the table

Next, Figure 2 shows the distribution of health professionals according to the stratification in the DCM quadrants, based on the division of the three dimensions proposed by the JSS: demand, control, and social support. The professionals surveyed have work that involves high psychological demand (66.0%), self-control (54.0%), and high social support (54.0%). By associating the analysis of the dimensions of the JSS, 18 (36.0%) were in high-demand work and five (10.0%) in passive work; 15 (30.0%) of the participants were in active work, with less risk to health. 

**Figure:**
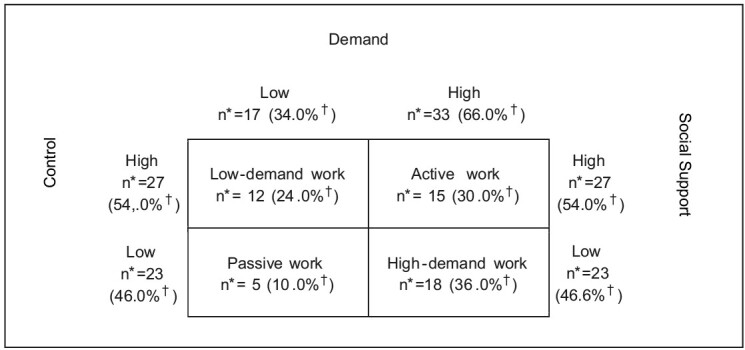
Figure 2 - Distribution of primary health care professionals during the COVID-19 pandemic, according to the quadrants of the Demand Control Model, according to the dichotomization of the Job Stress Scale dimensions (*n=50). Ijuí, RS, Brazil, 2021

*n = Sample; ^†^Percentage

### Self-perceived stress by PHC professionals during the COVID-19 pandemic: interfaces with demand, control, and social support

Through the qualitative approach, participants reported high work demands, resulting in high levels of exigency. There was an attempt to maintain self-control, being under high demand, and meeting the quantitative data. At the same time, they reported feelings such as anxiety, anguish, stress, insomnia, fear, insecurity, and vulnerability, highlighting the negative impacts on their physical and psychological well-being. However, data analysis revealed that most participants cited stress as the condition that established the most interfaces with their mental health, as shown in the statements:

*[...] I was very irritated; I was much more stressed. [...] more nervous at home, often taking it out on family members, [...] I cried a lot, so I felt it very strongly* (P2). 

*[...] external pressures brought me some stress that I did not expect. So much so that I needed to seek professional help, I couldn’t manage it alone* (P9). 

*It took me a while to realize that I was extremely stressed, [...] I answered everything very rudely, very nervously, and my stress level was very high* (P5). 

The number of workers under high demand stratified by DCM quadrants showed that most of them were under high psychological demand at work (66.0%). The interviews contributed to the understanding that these professionals’ work within PHC during the pandemic increased psychological demands and the risks of psychological harm:

*I was in a very tense moment. I was always irritated, nervous, working 10 hours a day [...] this increase in demand also affects us. It seems that we become incapable [...] I wanted to assist everyone and I assisted everyone. But then I got tense, stressed. [...] I have never had an experience like that* (P3). 

*[...] the struggle was: there are a lot of people sitting in the waiting room, who do I call first [...] with the start of vaccination campaigns there was another struggle [...] how to proceed, how to do it, how to schedule* (P2). 

*[...] I had to manage both COVID care and routine care [...] it changed the work routine very drastically and the workload which is already intense for nurses* (P5). 

Although 54.0% of the participants reported self-control, the high-demand work required a great effort from these professionals to maintain high control in the work environment and seek solutions to problems, which may favor greater exhaustion and increased complaints of psychological symptoms:

*In the beginning, everything was new. We didn’t know how we were going to do it, we were afraid we wouldn’t be able to do it, we wouldn’t be able to provide good care, we wouldn’t be able to make the diagnosis, we wouldn’t be able to treat. So, it was very difficult* (P13). 

*Because it is something new, you are in doubt, what you are going to do with that patient, you do not know where you are going to go. [...] you get anxious, you don’t know what to do, you go home wondering [...]. You can’t turn it off one hundred percent. These issues generate more insecurity, anxiety, and make you have concerns that you did not have before* (P1). 

*We feel that we are not meeting the demands that we should meet. This gradually affects our daily lives. When you can’t achieve the goals [...] everyone is overloaded* (P6). 

The perception of social support in the work environment was low for 46.6% of the participants. The interviews revealed that, despite being considered an important tool to minimize the risks for work-related stress development, almost half of the participants reported not feeling supported:

*[...] the administration tried, but they were not prepared for that either [...]. The municipality government does not offer psychological support [...] so I think there is a lack of support* (P11). 

*But I think there was a lack of support, suddenly even psychological support [...]. Because, although we were working, nobody thought about it. I think it was kind of left aside. Take care of the patient, and forget about the professional* (P1). 

Respondents also referred to the fear experienced in the work environment, as a result of COVID-19, as a factor of strong influence on their psychological health:

*[...] learn to work differently, learn to deal with the fear of assisting people, with the fear of becoming ill, of bringing illness into our homes* (P10). 

*It was very tough, the whole team was very scared, they were very afraid [...] we had restrictions, so we assisted less, but when we did, it was loaded with fear. Fear for us, for the patient, we always worked on the edge* (P2). 

*I was afraid, employees who were afraid, who started using psychotropic drugs and needed psychological and psychiatric monitoring. Employees said they did not sleep at night anymore, they were very afraid of being contaminated, of contaminating their families* (P5). 

In addition, health professionals reported a feeling of insecurity, vulnerability, and anxiety related to work, placing them as factors that interfered negatively with their mental health, highlighting the interface of these feelings with the increased psychological demand at work:

*In the beginning, it was very difficult, we got more scared I think, I even developed anxiety episodes, and I started taking medication* (P2). 

*I’ve never been so insecure. Very anxious, sometimes it seemed that I was having a panic attack [...]. Sometimes I want to go home. [...] I think I became a little more insecure in that sense. More afraid, more fearful* (P13). 

When integrating the data, interfaces were identified between high psychological demand and the presence of psychological damage symptoms, which can be explained by organizational changes and the particularities required at work, as well as by the perception of risks when working in the pandemic scenario. The results showed that part of the health professionals were subjected to highly demanding work during the pandemic. The reports of feelings of fear, vulnerability, insecurity, anxiety, and stress signaled that professionals were under high demand, which required a greater effort to maintain self-control and the self-balance necessary to suppress the high psychological demand in the face of the new work scenario. In addition, social support, which is a fundamental element to minimize risk factors for stress, was not perceived by almost half of the research participants. This may justify the fact that most of the interviewees present symptoms of occupational stress.

## Discussion

PHC health professionals are exposed to high risks of occupational stress. Individual characteristics, work, and professional category can contribute to the decrease or increase of such risks. This statement emerges from the reflections based on the results that showed that the highest percentage of participants claimed to have high psychological demands, yet they perceive self-control and high social support, minimizing the risks for occupational stress among them. The high score for high demand is consistent with what was brought up in the statements, in which the demands involved in the daily routine were observed in the participants’ reports.

Despite this, some professionals are still exposed to health risks. It was also found that the length of service and professional experience interfere with the way each person faces stressors. Another aspect that drew attention in this study is the fact that most professionals have a single employment contract, however, they feel psychologically exhausted and under high demand. This contradicts a study that showed multiple working hours as the main factor that interferes with the physical and mental health of professionals ^(^ 16 ^)^. Thus, it shows that the work during the pandemic required a great effort from these professionals to maintain their balance, favoring the emergence of several psychological symptoms, such as stress. 

Occupational stress is among the main causes of psychological distress during the pandemic ^(^ 17 ^)^ and can have negative consequences for workers, such as decreased quality of life and implications for mental health, high tension and sleep and emotional changes, impairing the workflow and the quality of care provided ^(^ 18 ^)^. 

The COVID-19 pandemic reflected intensely on primary care, causing profound changes in how care is provided and, as an infectious disease, presented an additional risk to health professionals who were subjected to high demands and with reduced teams, generating a work overload, favoring the emergence of factors that can trigger symptoms of physical and mental illness, leading to reduced concentration and reflecting on the quality of work ^(^ 19 ^)^. 

PHC health professionals are on the front line in the fight against COVID-19, considering PHC as the main gateway to the Unified Health System (UHS), it has as one of its attributions, to act directly in outbreaks and epidemics, developing a fundamental work in the fight against COVID-19 in Brazil ^(^ 20 ^)^. Professionals working in primary care during the pandemic expressed exhaustion and high risks of work-related stress and adverse conditions, which were added to their routine due to the pandemic ^(^ 21 ^)^. This study’s findings suggest that, during the pandemic, the psychological demands associated with the services provided in PHC were high and are among the main causes of psychological distress complaints. 

To better understand the relationships between occupational stress and work during the pandemic, the first analytical axis showed the demand, control, and social support interface of PHC professionals during the COVID-19 pandemic. The results obtained from the JSS showed that 66.0% of the participants were under high psychological demand, presenting a risk of exposure to stress in the work environment. These data are consistent with the qualitative stage, in which most respondents reported some degree of stress at work during the COVID-19 pandemic.

These findings were higher than the study with health professionals in Ethiopia, where the results revealed stress in 61.9% ^(^ 7 ^)^. Health professionals were especially affected by a high psychological burden related to the care process within health units. 

Of the total participants, 36.0% were in the high-demand work quadrant and 10.0% in the passive work quadrant. Thus, 46.0% of the workers were in a health risk situation, susceptible to the development of occupational stress. This has become common among health professionals and is considered a dangerous condition for the worker who, when exposed to situations with high demands, requires that the professional exceeds their individual and social capacity for coping, exceeding their personal resources, developing changes in the psychological state and generating emotional, cognitive, physiological and behavioral reactions ^(^ 22 ^)^. It can even result in a loss of skills and interest, which reflects negatively on health and the work environment ^(^ 12 ^)^. These consequences have already been observed in the participants’ statements during the interviews. 

30.0% of the professionals surveyed had active work and 24.0% had low-demand work. Active work occurs when high demands and self-control coexist, in this case, even if there are high demands, they are less harmful to health, while the individual has the possibility to plan their work according to their possibilities and create strategies to face it. These are considered the most favorable conditions for workers’ health ^(^ 23 ^)^. 

Despite the high psychological demand, most participants were in the self-control quadrant over work processes and high perception of social support. Self-control is related to the worker’s ability to use their skills to perform their work, as well as having the power to make decisions ^(^ 12 ^)^. Social self-support indicates good social interaction in the work environment, in addition to being considered a moderator of the factors that favor stress, and the lack of it can lead to negative consequences for the worker’s health ^(^ 24 ^)^. These results corroborate with a study in three public hospitals in Accra, Ghana, in which 40.11% of health professionals reported having adequate support from colleagues and management. In addition, 46.20% of the participants reported having the control and autonomy necessary to make decisions ^(^ 9 ^)^. 

Thus, the quantitative data and qualitative findings revealed that most participants reported high demand, however, they have control and high social support, which can favor the reduction of workers’ exposure to stressors, contributing to adaptation to demands and reducing the chances of psychological illness.

However, it is important to note that a considerable part of the sample studied is in the health risk quadrants, emphasizing the importance of mental health care for these professionals. In addition, work under high demand, added to the risk factors inherent to the pandemic, led to psychological overload, requiring a greater effort to maintain balance and control in the execution of activities. Working under these conditions favors the feelings that, over the course of the days, trigger physical and psychological illness ^(^ 25 ^)^. It is believed that, even with the end of the pandemic, the damage caused to mental health may remain and generate long-term harmful effects ^(^ 26 ^)^. 

The second analytical axis showed the interfaces of self-perceived stress by PHC professionals during the COVID-19 pandemic and DCM screening. Stressful events such as pandemics and natural disasters have a great impact on health professionals involved in care, because, while providing assistance to the population, they also experience these same impacts on their personal lives ^(^ 27 ^)^. This makes the health professional’s work environment even more loaded with stressors, which can have significant effects on their mental health ^(^ 21 ^)^. These repercussions were confirmed in the interviews, which revealed that the stressful factors resulting from the pandemic interfered with the quality of the professionals’ sleep and contributed to the increase in physical and mental fatigue. 

In parallel to the quantitative findings, the testimonies revealed that health professionals carry out their activities within PHC, subjected to feelings of fear, anxiety, insecurity, and vulnerability related to work in the pandemic scenario. Other studies have already identified these feelings among health professionals working in Hungary ^(^ 10 ^)^. Among these psychological reactions, fear was reported by 67.4% of participants in a study from Norway ^(^ 24 ^)^. Anxiety, depression, anguish, and insomnia in a Chinese study ^(^ 6 ^)^. These feelings are subjective to mental illness and have led to a greater psychological burden as a result of the pandemic ^(^ 10 ^)^. Through the testimonies, it is possible to understand that the exposure of these professionals to high demands and organizational changes in the primary care health sectors required greater effort in the search for adaptation to stressful factors, which may have contributed to the increase in the reported psychological symptoms. 

The interviews enabled the perception that specific factors related to the pandemic are placed as conditions that generate psychological distress, such as fear, insecurity, anxiety, and stress. In addition, the work of PHC during the pandemic contributed to these professionals feeling more insecure and vulnerable to occupational stress, such as exposure to the risk of infection, long working hours, psychological distress, fatigue, and professional exhaustion. It also identified the need for professional care and the use of medication to treat such symptoms, confirming psychological distress.

A statistically significant difference was found between DCM screening and occupation. The highest percentage of nursing and dentistry professionals were classified in the active labor force. These data differ from a study conducted with hospital care nurses in Iran, in which the physical and mental health of most nurses under high pressure and psychological stress, resulting from COVID-19, was impaired ^(^ 25 ^)^. This suggests that the responsibilities and duties of each occupation in different sectors may influence stressors in the work environment, contributing to aggravating or minimizing their harmful effects. 

Nutritionists and medical professionals, on the other hand, were classified in the high-demand quadrant, at high risk for the development of occupational stress. A study conducted with hospital doctors showed that they perceived counseling and social support to a greater extent when compared to nurses, in line with this study ^(^ 24 ^)^. This inequality in the results is related to hospital service structure and organizational aspects that differ when compared to the work developed in PHC. This study’s qualitative data contribute to the understanding that each profession’s responsibilities and attributions in care management act as a stressful factor in care during the COVID-19 pandemic. 

In this study, health professionals with more work experience were better able to deal with stressors, being more psychologically prepared to face the difficulties of working during the pandemic, when compared to those with less experience.

The association of quantitative and qualitative data showed that, according to the study participants’ perception, work during the pandemic had negative effects on the professionals’ psychological health. The high demands brought psychological overload, together with the lack of control and social support for some professionals, contributing to the emergence of factors that favored mental illness. Stressful factors have a great influence on the health and care provided, causing physical, psychological, social, and cultural damage, directly interfering with the quality of life and acting as the main causes of occupational stress ^(^ 28 ^)^. For the authors, the negative impact brought by stress goes beyond damage to physical and psychological health, also influencing the quality of their relationships, in the community and in the workplace. 

This study was limited by the fact that the interviews were conducted online, since face-to-face interaction allows for greater engagement and observation of body language. In addition, some procedures were not possible to strengthen the reliability of qualitative data - such as interview validation with the deponents, for example. The pandemic moment in which the data were collected was characterized by overload and exhaustion of frontline workers, which is why their availability to participate in the study was restricted.

The work of PHC health professionals during the COVID-19 pandemic can reflect negatively on their mental health, in addition to interfering with the quality of life and services provided. With effects that can go beyond the pandemic period. The results reinforce the associations between work variables and aspects related to physical and mental health, in synergy with the evidence produced in Brazil and other countries. However, the integration of findings from different sources of evidence and nature helped to advance the available knowledge until then, clarifying the implications of the pandemic on life, work, and interfaces with psychological symptoms.

Thus, this study contributed to the reflection on the importance of a healthy work environment within PHC and greater knowledge about the reality of the work scenario and the needs of these professionals, and can serve as a subsidy to health managers, to identify stressful factors in the work environment, collaborating to prevent health professionals’ mental illness.

## Conclusion

PHC health professionals worked under high psychological demands, with a high risk of exposure to occupational stress and psychological symptoms during the COVID-19 pandemic. Occupation and length of professional experience were associated with stress. On the other hand, the integration of these data with the qualitative findings showed the pandemic’s implications for life, work, and interfaces with psychological symptoms, strengthening the relationship between work in the pandemic and mental health.

This study showed that regardless of the work developed, health professionals were under high stress factors inherent to the work process during the pandemic. The results provide subsidies for understanding these professionals’ needs, signaling the importance of strategies aimed at strengthening PHC and caring for workers’ health in health institutions. Given their importance in primary care and that the negative effects on workers’ mental health also have repercussions in the post-pandemic period, strategies are needed to minimize such damage, promoting health, quality of life, and social support.
